# Genomic Insights into Selenate Reduction by *Anaerobacillus* Species

**DOI:** 10.3390/microorganisms13030659

**Published:** 2025-03-14

**Authors:** Qidong Wang, Jian Zhang, Jinhui Liang, Yanlong Wang, Chongyang Ren, Xinhan Chen, Dongle Cheng, Huanxin Zhang, Huaqing Liu

**Affiliations:** 1 College of Safety and Environmental Engineering, Shandong University of Science and Technology, Qingdao 266590, China; wang2000qd@163.com (Q.W.); zhangjian00@sdu.edu.cn (J.Z.); wangyanlong92@163.com (Y.W.); rency@sdust.edu.cn (C.R.); chenxinhan33@163.com (X.C.); donglelecheng@gmail.com (D.C.); liuhuaqings@163.com (H.L.); 2 Institute of Yellow River Delta Earth Surface Processes and Ecological Integrity, Shandong University of Science and Technology, Qingdao 266590, China; 3 School of Geographical Environment, Shandong Normal University, Jinan 250358, China; 4 State Environmental Protection Key Laboratory of Land and Sea Ecological Governance and Systematic Regulation, Jinan 250101, China; yellow2001hy@163.com; 5 Shandong Academy for Environmental Planning, Jinan 250101, China

**Keywords:** *Anaerobacillus* spp., metabolic versatility, selenium reduction, *serA*, gene clusters

## Abstract

Selenium (Se), a potentially toxic trace element, undergoes complex biogeochemical cycling in the environment, largely driven by microbial activity. The reduction in selenate or selenite to elemental selenium is an environmentally beneficial process, as it decreases both Se toxicity and mobility. This reduction is catalyzed by enzymes encoded by various related genes. The link between Se reduction gene clusters and specific taxonomic groups is significant for elucidating the ecological roles and processes of Se reduction in diverse environments. In this study, a new species of Se-reducing microorganism belonging to the genus *Anaerobacillus* was isolated from a mining site. A comparative analysis of the growth characteristics reveals that *Anaerobacillus* species exhibit notable metabolic versatility, particularly in their fermentation abilities and utilization of diverse electron donors and acceptors. Genome analysis identified a diverse array of gene clusters associated with selenate uptake (*sul*, *pst*), selenate reduction (*ser*), and selenite reduction (*hig*, *frd*, *trx*, and *bsh*). Since selenate reduction is the first crucial step in Se reduction, genes linked to selenate reductase are the focus. The *serA* gene clusters analysis suggests that the *serA* gene is highly conserved across *Anaerobacillus* species. The surrounding genes of *serA* show significant variability in both presence and gene size. This evolutionary difference in coenzyme utilization and *serA* regulation suggests distinct survival strategies among *Anaerobacillus* species. This study offers insights into Se bio-transformations and the adaptive strategies of Se-reducing microorganisms.

## 1. Introduction

Selenium (Se), a metalloid in Group VIA of the periodic table, is positioned between arsenic (As) and bromine (Br) in the fourth period. It presents contradictory ecological risks in the environment. Se is an essential component of various proteins known as selenoproteins or selenoenzymes, such as glutathione peroxidase and thioredoxin reductase [1,2]. Organisms ranging from microorganisms to plants and animals require trace amounts of Se, which is incorporated into specialized enzymes for essential metabolic functions [3]. However, Se is also a potent reproductive toxicant and teratogen, and excessive amounts can lead to toxicity in organisms [4]. Mining activities often result in elevated Se levels, causing contamination in surrounding environments. Such Se contamination poses significant threats to human health, wildlife, and microbial diversity [5].

The geochemical cycling and environmental behavior of Se is complex due to its multiple oxidation states (+VI, +IV, 0, and −II) and its presence in various forms, including solid, dissolved, and gaseous phases [6]. The speciation of Se in the environment is closely tied to prevailing redox conditions [7]. For example, the Se oxyanions, selenate (SeO_4_^2−^) and selenite (SeO_3_^2−^), are dominant in oxygenated environments [8]. These oxyanions can be reduced to elemental selenium (Se^0^) in anoxic and anaerobic conditions [9]. Selenium’s oxidation states directly affect its toxicity and mobility. SeO_3_^2−^ is more mobile than SeO_4_^2−^, while Se^0^ is considered immobile due to its low solubility. In terms of toxicity, Se species follow the order: SeO_3_^2−^ > SeO_4_^2−^ > Se^0^ [10]. In natural environments, Se transformations are largely driven by microbial processes, with microorganisms playing a crucial role in Se speciation, toxicity, and mobility [11,12].

The reduction of SeO_4_^2−^ or SeO_3_^2−^ to Se^0^ is an environmentally advantageous process, as it decreases both the toxicity and mobility of Se [13,14]. Both selenate and selenite serve as terminal electron acceptors during microbial respiration, a process linked to energy conservation. However, the microbial communities responsible for each process differ significantly [15]. For example, selenite was nearly stoichiometrically accumulated during selenate reduction by three isolates belonging to the genus *Pseudomonas*, indicating that these isolates are selenate reducers but not selenite reducers [16]. Additionally, *Rhizobium* sp. B1 demonstrated the ability to reduce selenite but not selenate [17]. These Se-reducing microorganisms are isolated from both Se-contaminated and uncontaminated environments. They belong to diverse phylogenetic groups such as Bacillota, Firmicutes, Proteobacteria, and Archaea [6,18,19]. Nevertheless, in comparison to As-reducing microorganisms, the knowledge regarding Se reducers remains limited.

The distinct microbial communities responsible for selenate and selenite reduction may be explained by the differences in the reductases required for each process. Selenate reduction is catalyzed by enzymes encoded by gene clusters such as *ser* [20], *srd* [21], *nap* [22], *ynf* [23], and *nar* [24]. In contrast, selenite reduction is facilitated by enzymes encoded by gene clusters including *srr* [25], *fcc* [26], *nir* [27], *gsr* [28], *trx* [29], *csr* [30], *hig* [31], *sir* [32], *ser* [33], *frd* [34], *bsh* [35], and *pit* [36]. Phylogenetically related bacterial species often share similar metabolic pathways, providing key insights into biogeochemical processes [37]. Understanding how Se reduction gene clusters or metabolic pathways are linked to specific taxonomic groups is essential for elucidating the ecological roles and mechanisms of Se reduction across different environments.

In this study, a Se-reducing microorganism from the genus *Anaerobacillus* was isolated from a mining environment. *Anaerobacillus* is a genus of spore-forming, anaerobic bacteria capable of surviving in extreme conditions and known for its ability to reduce metals and other compounds [38]. Notably, *Anaerobacillus* was identified as a significant genus of Se-reducing microorganisms. For example, *A. beveridgei*, *A. arseniciselenatis*, and *A. selenitireducens*, isolated from the arsenic-rich Mono Lake in California, can reduce both selenate and selenite [39,40,41]. In contrast, *A. macyae* JMM-4, isolated from an Australian gold mine, was shown to be incapable of reducing either selenate or selenite [42]. This study investigates the growth characteristics of Se-reducing *Anaerobacillus* species and explores their metabolic versatility through genomic profiling. The findings offer valuable insights into the Se-reduction mechanisms of *Anaerobacillus* species, with a particular focus on gene clusters responsible for encoding Se reductase.

## 2. Materials and Methods

### 2.1. Source of Inoculum and Preparation of Enrichment Cultures

Soil samples were collected aseptically from mining sites in Huaihua (110.04° E, 27.58° N), Enshi (109.48° E, 30.27° N), Xikuangshan (111.44° E, 27.69° N), Shimen (111.38° E, 29.58° N), Dushan (107.55° E, 25.82° N), and Nandan (107.54° E, 24.98° N). The samples were stored in sealed glass jars at −20 °C until further analysis. Enrichment cultures were prepared using a 10% (w/w) sediment inoculum, following strict anaerobic techniques, in minimal salt media containing 5 mM Na_2_SeO_4_ as the terminal electron acceptor and 10 mM acetate as the electron donor. The headspace was composed of a 70% CO_2_ and 30% N_2_ gas mixture, and 0.1 mM Na_2_S was added as a reducing agent [43]. Sterile controls were autoclaved at 121 °C for 20 min. Additionally, the controls without added substrates were included to assess background selenate reduction due to organic matter degradation in the sediment inoculum. All cultures were incubated in the dark at room temperature.

### 2.2. Isolation of Se-Reducing Microorganisms

Two isolation methods, selective nutrient media and colony picking from agar plates, were used to isolate Se-reducing bacteria from a complex microbial community [44]. Initially, cultures in selective selenate-containing media were used to enrich Se-reducing microorganisms. After two transfers in liquid media, 1 mL of the culture was transferred into 10 mL of agar media and serially diluted (10^−1^ to 10^−6^). After one week of incubation, distinct colonies were observed. Individual colonies with varying characteristics were selected and transferred to fresh agar media. This process was repeated multiple times to obtain pure cultures. Of the six soil samples, four (collected from Huaihua, Enshi, Shimen, and Nandan) exhibited SeO_4_^2−^ reduction activity. However, only one strain was successfully isolated from the Huaihua sample, and further analysis was conducted on this isolate. Based on phylogenetic consensus, this strain represents a novel species within the genus *Anaerobacillus*, for which the name *Anaerobacillus* sp. strain HL2 is proposed.

### 2.3. DNA Extraction and Genome Sequencing

Genomic DNA was extracted from 100 mL of aqueous culture using the phenol-chloroform method as previously described [45]. Briefly, 100 μL of Solution 1 (50 mM glucose, 10 mM EDTA, 25 mM Tris-Cl) was added to the samples, followed by five rapid freeze–thaw cycles between liquid nitrogen and a 55 °C water bath. Subsequently, 50 μL of 500 mM EDTA, 100 μL of lysozyme solution, and 150 μL of Solution 1 were added. The mixture was incubated at room temperature for 3–5 min. The samples were then extracted twice with 800 μL of phenol. The aqueous phase from each extraction was precipitated with ethanol in the presence of 2 μg of glycogen. DNA yields from the SeO_4_^2−^ culture were low, despite sufficient biomass. It is hypothesized that the Se^0^ nanoparticles produced during Se reduction may bind to the DNA, limiting extraction efficiency. The isolate was also capable of reducing arsenate to arsenite without forming particulate products. Culturing the isolate in arsenate medium allowed for successful DNA extraction (>400 ng) for genomic sequencing. The field sequencing kit (SQK-LRK001, Oxford Nanopore Technologies, Oxford, UK) was used to prepare the DNA library. The library was kept on ice until loaded into the flow cell (R9.4.1 revD, Oxford Nanopore Technologies). Read acquisition (MinKNOW core ver. 3.1.20) and base-calling (Guppy ver. 2.0.10) were preconfigured on the ONT MinIT device (Release 19.01.10). After 10 h of sequencing, the FASTQ files were submitted to MINDS ver. 1.0.53 for further analysis.

### 2.4. Genome Assembly, Annotation, and Analysis

The genome was deposited in the JGI/IMG database under Genome ID 2929886851. The sequence data were assembled using Velvet (version 0.7.63), and the consensus sequences were computationally shredded into overlapping synthetic reads. The assembled genomic DNA was annotated using PATRIC (version 3.6.9). The 16S rRNA sequence was extracted and analyzed via BLAST (MegAlign 7.0.26) in the NCBI database. Putative enzymes involved in selenate and arsenate reduction were identified by screening proteins with functional annotations. Pairwise average nucleotide identity (ANI) values between the whole genome sequence of the isolated strain and its closest relatives in the JGI database (https://img.jgi.doe.gov, accessed on 10 March 2025) were calculated. Additionally, *serA* gene clusters identified in the genomes of *Anaerobacillus* species were compared using the IMG/M system. A phylogenetic tree was constructed using MEGA 7.0, based on the *serA* gene sequence of the isolate and other *Anaerobacillus* species.

## 3. Results and Discussion

### 3.1. Growth Characteristic and Metabolic Diversity

**Figure 1:**
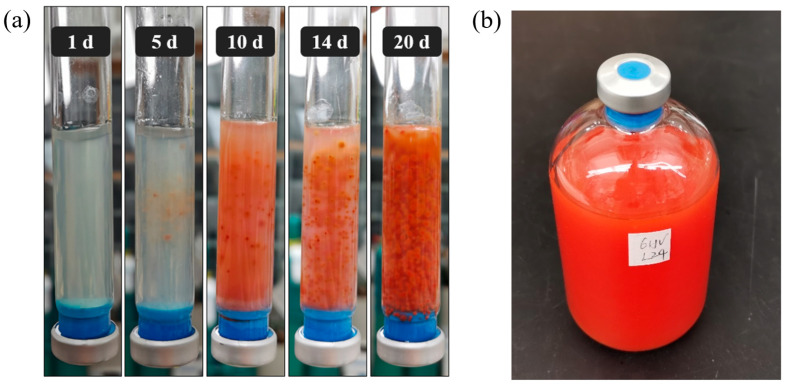
(**a**) Microbial reduction of SeO_4_^2−^ to Se^0^ over time by *Anaerobacillus* sp. strain HL2 isolate. (**b**) Transfer of culture into aqueous medium, demonstrating sustained metabolic activity in selenate reduction.

Microbial-induced selenate reduction refers to the process by which specific microorganisms convert SeO_4_^2−^, a highly soluble and mobile form of Se, into SeO_3_^2−^ and other less soluble forms, such as Se^0^ or Se^2−^. As demonstrated in Figure 1a, the isolate strain exhibits robust growth in anaerobic soft agar shake tubes, forming bright red colonies through the reduction of SeO_4_^2−^ to Se^0^. The produced Se^0^ is not further reduced to Se^2−^ by the isolate, as Se^2−^ does not display a uniform color and varies based on the specific metal or other elements it complexes with [46]. Se^0^ is a stable endpoint in many microbial reduction processes, and further reduction to Se^2−^ is uncommon, requiring specific conditions and potentially different microbial pathways [47]. In the microbial-mediated process, SeO_4_^2−^ acts as the electron acceptor, while acetate serves as the electron donor (Equation (1)). Upon transfer to aqueous media, the isolate maintained similar metabolic activity, demonstrating consistent selenate reduction (Figure 1b). This biochemical process is critical not only in the biogeochemical cycling of Se but also for environmental remediation. Microbial-induced selenate reduction can be applied to treat Se-contaminated mining runoff or industrial wastewater, mitigating selenium’s toxic effects on aquatic ecosystems when discharged into natural water bodies. Microbial Se^0^ has several distinct advantages that make it an environmentally friendly process over chemical approaches. Microbial routes typically generate nanoparticles with a well-defined size and morphology, which is advantageous in nanotechnology [48]. Moreover, microbial Se^0^ are usually biocompatible and are suited for medical application [49].

SeO_4_^2−^_(*aq*)_ + 2CH_3_COO^−^_(*aq*)_ + 4H_2_O_(*aq*)_ → Se^0^_(*s*)_ + 4HCO_3_^−^_(*aq*)_ + 10H^+^_(*aq*)_ (1)

The growth characteristics of *Anaerobacillus* sp. strain HL2 and eight other *Anaerobacillus* species were compared, focusing on their fermentation capabilities and utilization of various electron donors and acceptors (Table 1). Acetate was used by all strains except *A. beveridgei* MLTeJB, underscoring its general role as a key electron donor in this genus. Furthermore, it may boost the metabolic activity of the TCA cycle and fermentation pathways, potentially increasing the production of reducing equivalents that could enhance selenium reduction efficiency. In contrast, the utilization of starch and pyruvate varied across species, indicating their carbon metabolic diversity. Most *Anaerobacillus* species did not utilize oxygen, reflecting their predominantly anaerobic nature. However, *A. beveridgei* MLTeJB exhibited the unique ability to use oxygen, suggesting an evolutionary adaptation to both aerobic and anaerobic environments, which could be ecologically and biotechnologically significant. The metabolic versatility of the *Anaerobacillus* genus is further demonstrated by their ability to use nitrate, selenate, selenite, and arsenate as electron acceptors. Notably, *A. selenitireducens* MLS10 and *A. alkalidiazotrophicus* MS 6 can reduce nitrate, facilitating survival in nitrogen-rich environments and contributing to denitrification, a key process for mitigating nitrate pollution. Five species, including *Anaerobacillus* sp. strain HL2, *A. selenitireducens* MLS10, *A. beveridgei* MLTeJB, *A. alkalilacustris* Z-05211, and *A. arseniciselenatis* E1H, have demonstrated the ability to reduce selenate, while *A. macyae* JMM-4 and *A. alkalidiazotrophicus* MS 6 do not. Importantly, all selenate-reducing species can also reduce selenite. The ability to utilize arsenate varied among the strains, reflecting specific environmental adaptations. These differences in electron acceptor usage highlight the metabolic flexibility of *Anaerobacillus* species, enabling them to survive and function in diverse and often challenging environmental conditions. Furthermore, metabolomic analyses could further elucidate how different electron donors influence metabolic pathways driving selenium reduction, offering a promising direction for future research.

### 3.2. Comprehensive Genome Profiling of Anaerobacillus Species

#### 3.2.1. Overall Genome Characteristic

**Figure 2:**
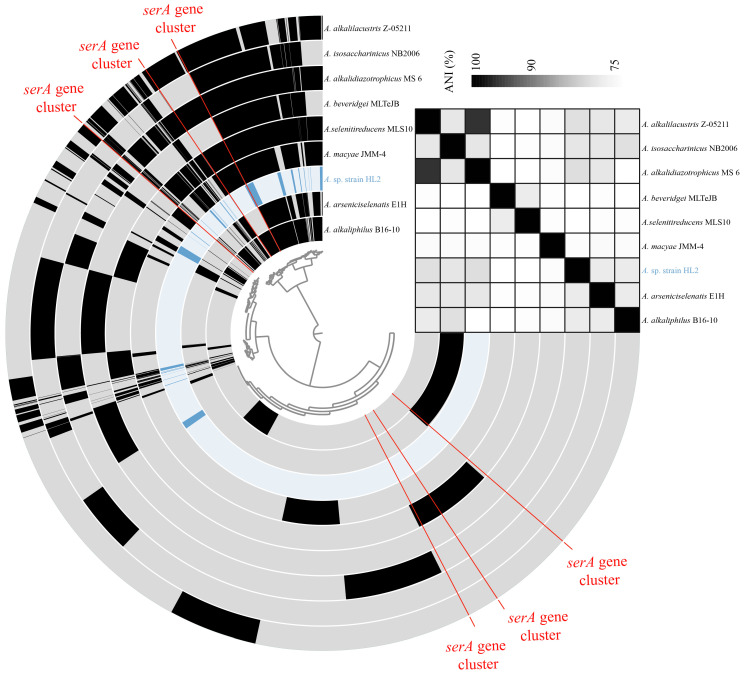
The pangenomic analysis of different *Anaerobacillus* species. The darker colors indicate the presence of gene clusters in each genome. The red lines connected to the center of the graph indicate the positions of the *serA* gene clusters. The heat map represents the average nucleotide identity (ANI) between each pair of different *Anaerobacillus* species.

The genome of *Anaerobacillus* sp. strain HL2 comprises 3.6 Mb with a GC content of 35.0%. Within the genome, 10,686 coding sequences are present, including 81 tRNA and 21 rRNA genes. Specifically, there are six copies of 5S rRNA, nine copies of 16S rRNA, and six copies of 23S rRNA. The sequence similarity of the nine copies of 16S rRNA exceeds 99%. The genome also contains 7503 hypothetical proteins and 3183 proteins with functional annotations (Table S1, Figure S1). Figure 2 presents a pairwise comparison of the average nucleotide identity (ANI) values among different *Anaerobacillus* species. ANI values serve as a measure of genomic similarity, with a threshold of 95% commonly used to classify organisms as the same species [55]. Notably, only one comparison, between *A. alkalilacustris* Z-05211 and *A. alkalidiazotrophicus* MS 6, exhibits an ANI value above 95% (95.3%), indicating these two isolates belong to the same species. All other comparisons show ANI values below 95%, suggesting that the respective species are genetically distinct. For instance, all ANI comparisons involving *Anaerobacillus* sp. strain HL2 and other *Anaerobacillus* species fall below the 95% threshold, supporting the identification of *Anaerobacillus* sp. strain HL2 as a novel species within the genus. Overall, the consistently low ANI values across most species pairs highlight the genetic diversity within the *Anaerobacillus* genus.

#### 3.2.2. Identification of Genes Related to Se Transport and Transformation

**Figure 3:**
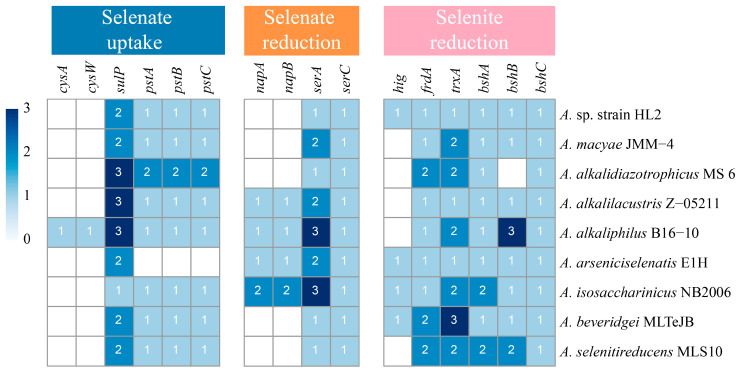
Number of putative genes involved in Se transport and transformation identified in *Anaerobacillus* sp. strain HL2.

A summary of the identified genes involved in Se transport and transformation across various *Anaerobacillus* species is provided, with a focus on the presence and copy number of genes related to selenate uptake, selenate reduction, and selenite reduction (Figure 3). Key genes, such as *cysAW*, *sulP*, and *pstABC*, are highlighted in the context of selenate uptake. The first set of genes, the *cys* gene cluster, is primarily responsible for sulfur compound transport, yet it also facilitates the uptake of selenate due to the structural similarity between sulfate and selenate. Specifically, *cysW* encodes a membrane protein involved in the sulfate-thiosulfate transporter system, while *cysA* encodes an ATP-binding protein that powers selenate uptake via ATP hydrolysis [56]. This transport mechanism, driven by the *cys* gene cluster, is observed in *A. alkaliphilus* B16-10, suggesting its importance in selenate uptake within this species. The second gene involved is *sulP*, which encodes sulfate permease (SulP), a protein that mediates the transport of sulfate and similar anions, including selenate [57]. Competitive inhibition studies in *Desulfovibrio desulfuricans* CSN indicate that sulfate and selenate share a SulP-mediated transport pathway [58]. Further evidence from *Arabidopsis thaliana* mutants, which show reduced selenate uptake when the *sulP* gene is disrupted, underscores the gene’s role in selenate transport [59]. The ubiquitous presence of *sulP* in all *Anaerobacillus* species suggests that this gene plays a critical role in selenate uptake across the genus. Finally, the *pst* gene cluster encodes the high-affinity phosphate transport system (Pst system), which includes *pstA*, *pstB*, and *pstC* genes. Due to the structural similarity between phosphate and selenate, the Pst system also facilitates selenate uptake, particularly via PstA [36]. However, in phosphate-rich environments, phosphate transport is prioritized, limiting selenate uptake. Interestingly, *pstA*, *pstB*, and *pstC* genes are present in most *Anaerobacillus* species, except for *A. arseniciselenatis* E1H, suggesting that the *pst* gene cluster may have an alternative or additional role in selenate transport in the majority of *Anaerobacillus* species.

Selenate reduction-related genes in the *nap* and *ser* clusters were identified in the genomes of *Anaerobacillus* species (Figure 3). Although *srdA*, *ynfE*/*ynfF*, and *narG* are recognized as functional genes in the selenate reduction process, these genes are not present in all *Anaerobacillus* species [21,24,60]. The *napA* and *napB* genes encode the subunits of the periplasmic nitrate reductase (Nap) enzyme, with NapA serving as the molybdenum-containing catalytic subunit and NapB as a diheme cytochrome c involved in electron transfer. Predominantly found in denitrifies like *Rhodobacter sphaeroides*, Nap plays a key role in the initial step of denitrification. While NapA and NapB can reduce selenate and tellurite in vitro, their activities toward these oxyanions are significantly less efficient compared to nitrate reduction [22]. The presence of the *napA* and *napB* genes in *A. alkalilacustris* Z-05211, *A. alkaliphilus* B16-10, *A. arseniciselenatis* E1H, and *A. isosaccharinicus* NB2006 suggests that the Nap enzyme may play a role in selenate reduction in these species. On the other hand, the *ser* genes, including *serA*, *serB*, and *serC*, encode the subunits of the periplasmic selenate reductase in *Thauera selenatis* [61]. *serA* encodes the molybdenum-containing catalytic subunit, which is proposed to host the active site for selenate reduction [62]. *serB* encodes an iron-sulfur protein, which likely participates in electron transfer within the enzyme complex. Its structure includes cysteine residues typical of iron-sulfur clusters, suggesting its role in mediating electron flow to the catalytic SerA subunit. Finally, SerC, the smallest subunit, is hypothesized to contain a heme b component, similar to cytochromes, potentially facilitating electron transfer from SerB to SerA during the reduction process [63]. The *ser* gene cluster is present in all *Anaerobacillus* species, underscoring its significant role in selenate reduction.

Selenite reduction occurs as a subsequent process following the reduction in selenate to selenite. The related genes, including *hig*, *frdA*, *traA*, and *bshABC*, were identified in the genomes of *Anaerobacillus* species (Figure 3). The gene *hig* encodes Hydrogenase I, which functions as a selenite reductase in *Clostridium pasteurianum*. The purified enzyme demonstrated the ability to reduce SeO_3_^2−^ to Se^0^ through the oxidation of H_2_. The observed stoichiometry of 2.3:1 (H_2_:Se^0^) closely approximates the theoretical ratio of 2:1, confirming the high efficiency of the reduction process [31]. Hydrogenase I has not been reported to catalyze selenite reduction using organic electron donors, despite the identification of the *hig* gene in certain *Anaerobacillus* species. The second gene of interest is *frdA*, which encodes fumarate reductase, an enzyme essential for selenite reduction via a membrane-bound mechanism. Proteomic and genetic analyses reveal that selenite exposure significantly upregulates fumarate reductase expression in *Enterobacter cloacae* Z0206. Knockout experiments further confirm the enzyme’s crucial role, as deletion of *frdA* markedly impairs the bacterium’s ability to reduce selenite [34]. The third gene involved is *trxA*, which encodes thioredoxin. Thioredoxin is a key component of the thioredoxin system and mediates redox reactions. These reactions include the reduction in selenite. In *Pseudomonas stutzeri*, *trxA* plays a critical role in the initial step of selenite reduction. Both *frdA* and *trxA* genes are found in all *Anaerobacillus* species, suggesting their potential role in selenite reduction. The *bsh* gene cluster is another set of genes associated with selenite reduction [35]. This cluster, which includes *bshA*, *bshB*, and *bshC*, is involved in the biosynthesis of bacillithiol, an important low-molecular-weight thiol found in Gram-positive bacteria, particularly in *Bacillus* species. Specifically, *bshA* encodes N-acetyl-alpha-D-glucosaminyl L-malate synthase, *bshB* encodes a deacetylase, and *bshC* encodes a cysteine-adding enzyme. Together, these genes facilitate the production of bacillithiol, which plays a key role in selenite reduction by forming selenodibacillithiol intermediates. These intermediates are further reduced to Se^0^ through enzymatic processes, including the thioredoxin and thioredoxin reductase systems, contributing to microbial selenite reduction. As is consistent with the presence of *frdA* and *trxA* genes, the *bsh* gene clusters are found in all *Anaerobacillus* species, highlighting the metabolic versatility of the selenite reduction process.

#### 3.2.3. Comparative Analysis of *serA* Gene Cluster in *Anaerobacillus* Species

Although gene clusters related to Se reduction were identified, certain *Anaerobacillus* species can reduce Se, while others cannot. This highlights the need for a deeper investigation into their molecular mechanisms. As the reduction in selenate is a critical initial step in Se reduction, genes associated with selenate reductase have become a central focus of this study. The *ser* gene cluster, specifically linked to selenate reduction, includes *serA*. This gene is reported to contain the active site responsible for the reaction [62]. Therefore, further analysis of the *serA* gene cluster in *Anaerobacillus* species is necessary to elucidate its distinct role and functionality.

**Figure 4:**
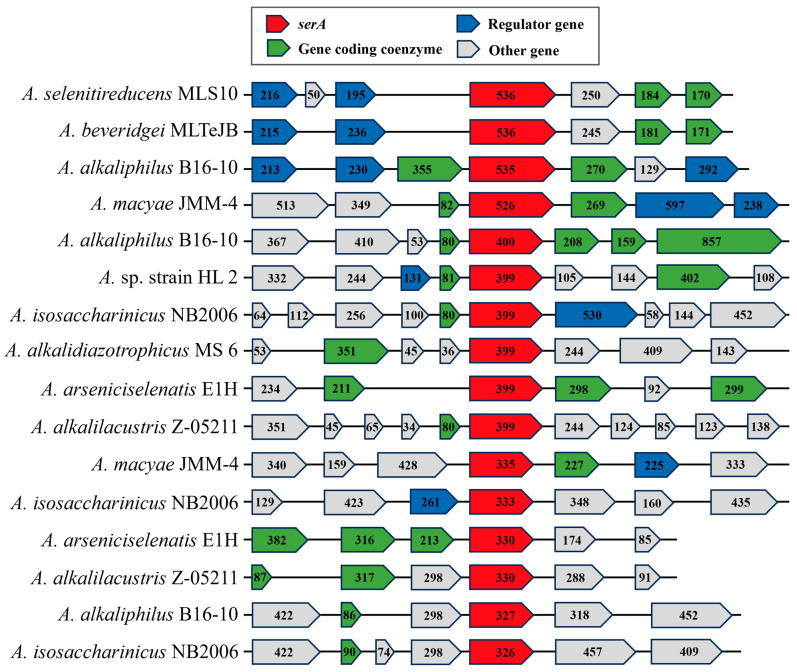
Comparison of *serA* gene clusters in strain HL2 and related *Anaerobacillus* species. Numbers indicate amino acid (aa) values of respective genes.

A comparison of the *serA* gene clusters in the *Anaerobacillus* species reveals both conserved and divergent patterns in gene organization (Figure 4). Among anaerobic *Anaerobacillus* species, the *serA* gene remains highly conserved, exhibiting only slight differences in amino acid length. In contrast, the regulatory elements and coenzyme-related genes adjacent to *serA* display considerable variability in both their presence and size. This variation likely reflects species-specific adaptations within *Anaerobacillus*, indicating distinct regulatory or metabolic mechanisms associated with *serA*. Specifically, two regulatory genes are located upstream of the *serA* gene in *A. selenitireducens* MLS10, *A. beveridgei* MLTeJB, and *A. alkaliphilus* B16-10. One set of these genes belongs to the Crp/Fnr and GntR families, which are key bacterial transcriptional regulators involved in metabolic and environmental response pathways. Fnr, specifically, controls anaerobic respiration by modulating reductase enzymes. This enzyme is critical in electron transport, enabling bacterial adaptation to oxygen-limited environments [64]. The second regulatory gene belongs to the GntR family, which primarily regulates carbohydrate metabolism, often functioning as a repressor [65]. Moreover, three other downstream genes encode enzymes: histidinol-phosphatase, ATP(I)alamin adenosyl transferase, and molybdopterin adenylyl transferase, respectively. Histidinol-phosphatase catalyzes the dephosphorylation of histidinol phosphate to histidinol, a key step in histidine biosynthesis [66]. ATP(I)alamin adenosyl transferase is crucial for converting cob(I)alamin to adenosyl cobalamin (coenzyme B12), a cofactor involved in reductase reactions such as methyl malonyl-CoA mutase activity in propionate metabolism [67]. Molybdopterin adenylyl transferase is essential for the biosynthesis of molybdenum cofactors, which are required by molybdenum-containing reductases that participate in electron transport [68]. These enzymes play a vital role in supporting reductase activity by facilitating cofactor biosynthesis and metabolic processes. The *fer* gene, which encodes the ferredoxin protein, is present in many species of *Anaerobacillus*. Ferredoxin, a small iron-sulfur protein, plays a crucial role in electron transfer during various biological redox reactions. Acting as an electron carrier, ferredoxin facilitates the transfer of electrons to a variety of reductases, driving enzymatic reduction processes [69]. Numerous unannotated genes are located both upstream and downstream of the *serA* gene, suggesting that further investigation using an integrated approach of proteomic analysis and gene knockout experiments is needed to explore the relationship between this gene cluster and selenate reduction. Notably, some gene clusters, such as the *serA* clusters in *Anaerobacillus macyae* JMM-4 and *Anaerobacillus alkalidiazotrophicus* MS 6, do not appear to contribute to selenate reduction (Table 1). These findings highlight the evolutionary divergence in the mechanisms regulating coenzyme utilization and *serA* expression across *Anaerobacillus* species.

The phylogenetic relationships of *serA* genes from various *Anaerobacillus* species were analyzed (Figure S2). Notably, branch lengths in the phylogenetic tree correspond to genetic distance, with longer branches indicating greater genetic divergence. While all species harbor a common *serA* gene, the degree of sequence similarity varies, highlighting evolutionary divergence within the *Anaerobacillus* genus. For example, the *serA* gene similarity between *A. selenitireducens* MLS10 and *A. beveridgei* MLTeJB is 76% (Figure S3). Proteins with over 70% sequence identity have at least a 90% chance of being involved in the same biological process. The *serA* genes of *A. alkalilacustris* Z-05211 and *A. alkalidiazotrophicus* MS 6 exhibit the highest similarity (96%), a phenomenon consistent with the ANI of their genomes (Figure 2). The phenomenon further suggests that closely related bacterial species often share similar metabolic processes.

#### 3.2.4. Metabolic Pathway Linking Se Reduction in *Anaerobacillus* sp. Strain HL2

**Figure 5:**
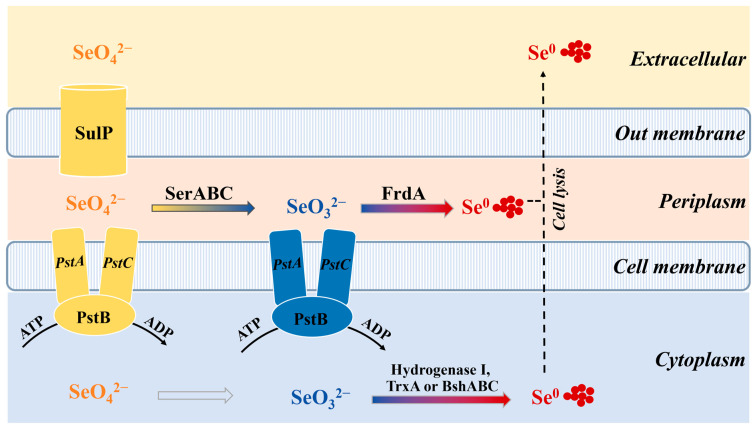
Proposed Se metabolic pathway in *Anaerobacillus* sp. strain HL2.

Based on the Se transport and transformation genes identified in *Anaerobacillus* sp. strain HL2, the proposed metabolic pathways for Se reduction by strain HL2 are illustrated in Figure 5. Selenate is taken up by SulP transporters and reduced to selenite potentially by an enzymatic complex labeled SerABC. Further reduction occurs in the periplasm, where FrdA facilitates the conversion of selenite. The selenite can then be transported to the cytoplasm by PstABC, where it is further reduced to Se^0^ by enzymes such as Hydrogenase I, TrxA, or BshABC [36]. A question mark in the diagram highlights an unknown or unidentified enzyme in the Se(VI) to Se(IV) reduction pathway within the cytoplasm. The model also suggests that Se^0^ may be released extracellularly, possibly through cell lysis, though specific transmembrane mechanisms remain unidentified. This model provides insights into Se detoxification pathways in microbial systems, with implications for bioremediation of Se-contaminated environments.

## 4. Conclusions

A novel *Anaerobacillus* species capable of reducing selenate and selenite was isolated from a mining site. Comparative growth studies indicate the metabolic versatility of *Anaerobacillus* species, particularly their ability to utilize diverse electron donors and acceptors. The gene clusters associated with selenate uptake, selenate reduction, and selenite reduction are identified by genomic analysis. A high degree of conservation of the *serA* gene across *Anaerobacillus* species is observed. The regulatory and coenzyme-related genes in the surrounding regions are distinct. This divergence in coenzyme utilization and *serA* regulation suggests different survival strategies and evolutionary pathways of these *Anaerobacillus* species. The Se transformation mechanisms could be further revealed by employing proteomic analysis in combination with gene knockout experiments to characterize the expression of *serA*, *serB*, and *serC* and to uncover unexploited functions associated with these proteins. Although *Anaerobacillus* species exhibit high efficiency in selenium bio-transformation, their environmental adaptability and stability need to be further considered for practical remediation applications.

## Figures and Tables

**Table 1:** Comparative growth characteristics of *Anaerobacillus* sp. strain HL2 and related species within genus *Anaerobacillus*. Strains compared are as follows: 1. *Anaerobacillus* sp. strain HL2; 2. *A. macyae* JMM-4; 3. *A. selenitireducens* MLS10; 4. *A. beveridgei* MLTeJB; 5. *A. alkalidiazotrophicus* MS 6; 6. *A. alkalilacustris* Z-05211; 7. *A. arseniciselenatis* E1H; 8. *A. alkaliphilus* B16-10; and 9. *A. isosaccharinicus* NB2006.

Metabolism	1	2 ^a^	3 ^b^	4 ^c^	5 ^d^	6 ^e,f^	7 ^a^	8 ^g^	9 ^h^
Fermentation	−	−	+	+	+	+	+	−	+
Electron Donors									
Acetate	+	+	+	−	+	+	−	−	−
Starch	+	−	+	+	+	+	+	−	+
Pyruvate	−	+	+	+	n.a.	+	−	−	+
Electron Acceptors									
Oxygen	−	−	−	+	−	−	−	+	−
Nitrate	−	−	+	+	−	−	+	+	+
Selenate	+	−	+	+	−	+	+	n.a.	n.a.
Selenite	+	−	+	+	−	+	+	n.a.	n.a.
Arsenate	+	+	+	+	−	+	+	−	+

Note: a, data from [42]; b, data from [41]; c, data from [40]; d, data from [50]; e, data from [51]; f, data from [52]; g, data from [53]; h, data from [54]. +, positive; −, negative; n.a., no data available.

## Data Availability

The genome was deposited in the JGI/IMG database under Genome ID 2929886851.

## Supplementary Materials

The following supporting information can be downloaded at: https://www.mdpi.com/article/10.3390/microorganisms13030659/s1. Figure S1: Circular representation of genome characteristic of *Anaerobacillus* sp. strain HL2; Figure S2: Phylogenetic tree of *serA* genes from *Anaerobacillus* species was constructed using Maximum Likelihood method based on Tamura–Nei model. Analysis included 16 nucleotide sequences, with all gaps and missing data excluded, resulting in final dataset of 910 positions; Figure S3: The similarity of the identified *serA* genes in the *Anaerobacillus* species. The red numbers represent the corresponding amino acid (aa) values for each gene; Table S1: General features of annotated genome *Anaerobacillus* sp. strain HL2.
