# Supplementary material for: Genomic Insights into Selenate Reduction by Anaerobacillus Species
Source: Microorganisms. 2025 Mar 14;13(3):659. doi: 10.3390/microorganisms13030659 (PMC11944866; doi:10.3390/microorganisms13030659)
Supplement: Supplementary file 1 [file microorganisms-13-00659-s001.zip › microorganisms-3511536-supplementary.pdf]

Supporting Information

Genomic Insights into Selenate Reduction by *Anaerobacillus* Species

Qidong Wang[1, 2], Jian Zhang[1, 2, 3], Jinhui Liang[4, 5], Yanlong Wang[1, 2], Chongyang Ren[1, 2], Xinhan Chen[1, 2], Dongle Cheng[1, 2], Huanxin Zhang[1, 2, *], Huaqing Liu[1, 2]

1 College of Safety and Environmental Engineering, Shandong University of Science and Technology, Qingdao 266590, China
2 Institute of Yellow River Delta Earth Surface Processes and Ecological Integrity, Shandong University of Science and Technology, Qingdao 266590, China
3 School of Geographical Environment, Shandong Normal University, Jinan 250358, China
4 State Environmental Protection Key Laboratory of Land and Sea Ecological Governance and Systematic Regulation, Jinan 250101, China
5 Shandong Academy for Environmental Planning, Jinan 250101, China
* Correspondence: liuhuaqing@sdust.edu.cn

The Supporting Information contains detailed materials and methods, 1 tables and 3 figures.

Table S1. General features of the annotated genome *Anaerobacillus* sp. strain HL2…………..…P3

Figure. S1. Circular representation of the genome characteristic of *Anaerobacillus* sp. strain HL2..……………………………………………………………………………………….P4

Figure. S2. Phylogenetic tree of *serA* genes from *Anaerobacillus* species was constructed using the Maximum Likelihood method based on the Tamura-Nei model. The analysis included 16 nucleotide sequences, with all gaps and missing data excluded, resulting in a final dataset of 910 positions. .P5

Figure. S3 The similarity of identified *serA* genes in *Anaerobacillus* species. The red numbers represent the corresponding amino acid (aa) values for each gene…………………………………P6

Table S1. General features of the annotated genome *Anaerobacillus* sp. strain HL2.

**Sequence Info**	**Values**
Contigs	1
Genome length	3,701,057
GC content	35.0%
**Genomic features**	**Values**
Coding sequence	10686
tRNA	81
rRNA	21
5S rRNA	6
16S rRNA	9
23S rRNA	6
**Protein features**	**Values**
Hypothetical proteins	7503
Proteins with functional assignments	3183

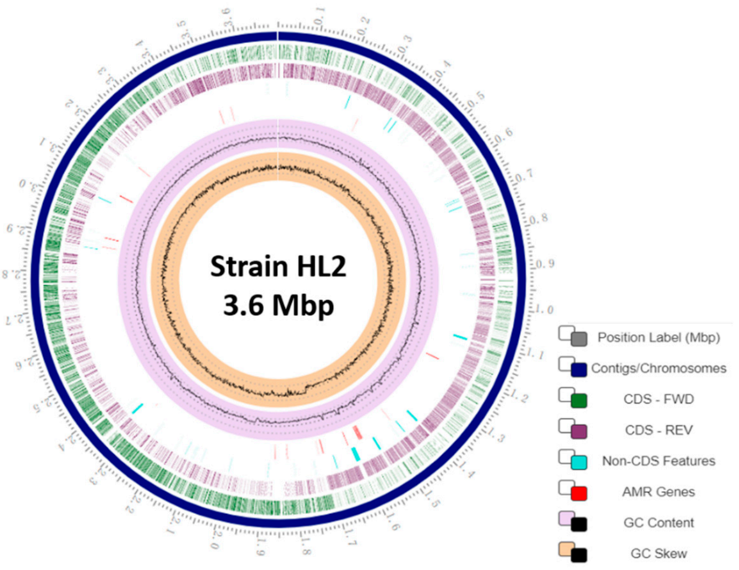

Figure. S1. Circular representation of the genome characteristic of *Anaerobacillus* sp. strain HL2.

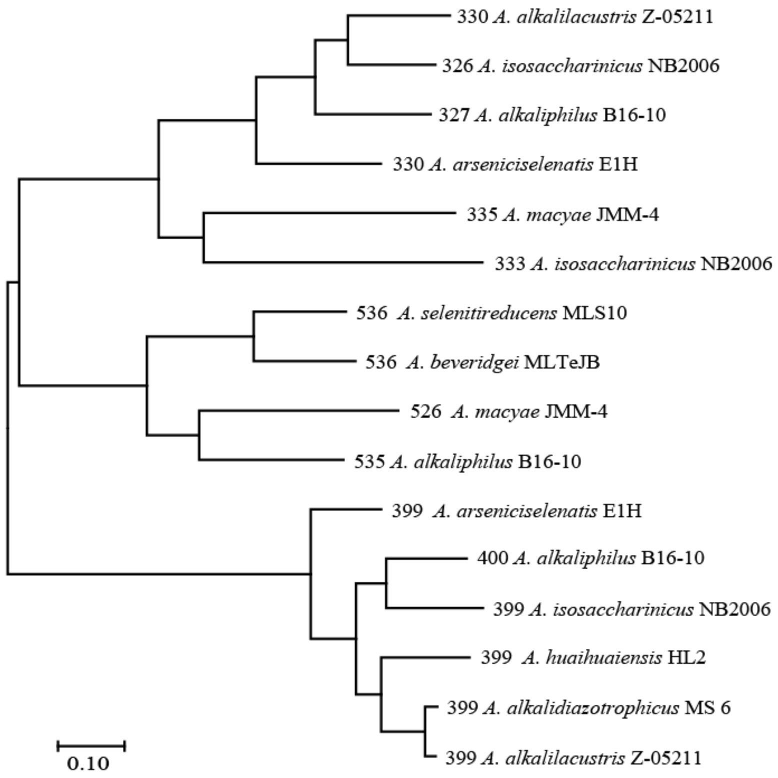

Figure. S2. Phylogenetic tree of *serA* genes from *Anaerobacillus* species was constructed using the Maximum Likelihood method based on the Tamura-Nei model. The analysis included 16 nucleotide sequences, with all gaps and missing data excluded, resulting in a final dataset of 910 positions.

	536 *A. selenitireducens* MLS10	526 *A. macyae* JMM-4	335 *A. macyae* JMM-4	399 *A. arseniciselenatis* E1H	330 *A. arseniciselenatis* E1H	399 *A. alkalidiazotrophicus* MS 6	330 *A. alkalilacustris* Z-05211	399 *A. alkalilacustris* Z-05211	536 *A. beveridgei* MLTeJB	399 *A.* sp. strain HL2	327 *A. alkaliphilus* B16-10	535 *A. alkaliphilus* B16-10	400 *A. alkaliphilus* B16-10	326 *A. isosaccharinicus* NB2006	333 *A. isosaccharinicus* NB2006	399 *A. isosaccharinicus* NB2006
536 *A. selenitireducens* MLS10																
526 *A. macyae* JMM-4	58%															
335 *A. macyae* JMM-4	42%	43%														
399 *A. arseniciselenatis* E1H	45%	46%	49%													
330 *A. arseniciselenatis* E1H	42%	44%	58%	48%												
399 *A. alkalidiazotrophicus* MS 6	47%	47%	50%	77%	48%											
330 *A. alkalilacustris* Z-05211	42%	43%	59%	49%	70%	50%										
399 *A. alkalilacustris* Z-05211	46%	48%	50%	78%	48%	96%	50%									
536 *A. beveridgei* MLTeJB	76%	58%	44%	48%	42%	46%	42%	46%								
399 *A.* sp. strain HL2	46%	48%	50%	76%	48%	80%	49%	80%	47%							
327 *A. alkaliphilus* B16-10	42%	43%	56%	48%	68%	49%	73%	50%	41%	49%						
535 *A. alkaliphilus* B16-10	61%	63%	42%	46%	43%	49%	40%	47%	61%	46%	42%					
400 *A. alkaliphilus* B16-10	47%	48%	50%	76%	48%	78%	49%	78%	47%	76%	48%	47%				
326 *A. isosaccharinicus* NB2006	40%	43%	58%	48%	70%	49%	76%	49%	42%	48%	74%	43%	48%			
333 *A. isosaccharinicus* NB2006	43%	43%	55%	49%	57%	49%	57%	50%	43%	50%	56%	43%	50%	58%		
399 *A. isosaccharinicus* NB2006	47%	48%	50%	75%	49%	77%	49%	77%	46%	77%	50%	48%	78%	49%	50%	

Figure. S3. The similarity of identified *serA* genes in *Anaerobacillus* species. The red numbers represent the corresponding amino acid (aa) values for each gene.
